# Ethnic Differences in Personality Disorder Patterns among Women Veterans Diagnosed with PTSD

**DOI:** 10.3390/bs4010072

**Published:** 2014-03-10

**Authors:** Janet C’de Baca, Diane T. Castillo, Julia E. Mackaronis, Clifford Qualls

**Affiliations:** 1 New Mexico VA Health Care System, Albuquerque, New Mexico, 1501 San Pedro SE, Albuquerque, NM 87108, USA; E-Mails: Diane.Castillo@va.gov (D.T.C.); julia.mackaronis@gmail.com (J.E.M.); 2 Department of Psychiatry, School of Medicine, University of New Mexico, MSC 09 5030, 1 University of New Mexico, Albuquerque, NM 87131; USA; 3 Department of Psychology, University of Utah, 380 South 1530 East, BEHS 502, Salt Lake City, UT 84112, USA; 4 Clinical and Translational Science Center (CTSC), University of New Mexico, MSC 09 5030, 1 University of New Mexico, Albuquerque, NM 87131, USA; E-Mail: cqualls@salud.unm.edu

**Keywords:** personality disorders, PTSD, women, veterans, race, ethnicity

## Abstract

Personality Disorders (PDs) impair the ability to function socially and occupationally. PD prevalence rates among veterans who have also been diagnosed with posttraumatic stress disorder (PTSD) range from 45%–79%. This study examined ethnic differences in PDs assessed with the Millon Clinical Multiaxial Inventory-III in 260 non-Hispanic white (64%), Hispanic (27%), and African American (9%), mostly single, women veterans in treatment for PTSD. After adjusting for covariates including number and sexual-nature of trauma, findings revealed the adjusted odds ratio of having a cluster A PD was almost three times higher for African Americans (*p* = 0.046) then the other two ethnic groups, which may be driven by the paranoid PD scale and potentially reflects an adaptive response to racial discrimination. In cluster designation analysis, the odds were twice as high of having a cluster B PD with childhood trauma (*p* = 0.046), and a cluster C PD with sexual trauma (*p* = 0.004), demonstrating the significance of childhood and sexual trauma on long-term chronic personality patterns in women veterans. These results highlight the importance of using instruments with demonstrated diagnostic validity for minority populations.

## 1. Introduction

Personality Disorders (PDs) are inflexible patterns of perceiving, reacting, and relating to self, others, and the environment, and often cause considerable impairment in the ability to function socially [1] and occupationally [2]. In the DSM-5 [1], personality disorders are grouped into three clusters, with each cluster based on overall similarities in terms of cognitive, emotional, and behavioral patterns. Cluster A includes paranoid, schizoid, and schizotypal PDs; cluster B antisocial, borderline, histrionic, and narcissistic PDs; and cluster C avoidant, dependent, and obsessive-compulsive PDs. General population prevalence rates are 5.7% for cluster A, 1.5% cluster B, and 6.0% cluster C [3].

A growing literature indicates PDs are highly comorbid with Posttraumatic Stress Disorder (PTSD) [4,5,6]. For example, Friborg, Martinussen, Kaiser, Overgard, and Rosenvinge [7] reported a 35% comorbidity rate for PTSD and PDs. Overall, studies of veterans suggest rates of comorbid PTSD and PDs are as high as 45% in outpatient and 79% in inpatient samples [8,9].

Soldiers experiencing combat or sexual trauma are at the highest risk for developing PTSD [10,11,12]. Kang, Dalager, Mahan, and Ishii [13] reported the adjusted odds ratio for PTSD associated with military sexual trauma at 5.41 and for high combat exposure at 4.03 for women veterans. Data from the Veterans Affairs (VA) [14] universal screening program revealed 1 in 5 females (20%) and 1 in 100 (1%) male veterans receiving care at the VA experienced sexual trauma during their military service. Additionally, the number of women in the military is on the rise [15], and, in 2010 about half were minorities; African American women made up 31%, Hispanic women 13%, and multiracial women 7% of enlisted active forces [16].

The prevalence of PDs by ethnicity is unclear. McGilloway, Hall, Lee, and Bhui’s [17] meta-analysis of prevalence studies indicates lower prevalence of PDs among African Americans compared to non-Hispanic whites, and no difference between Hispanics and non-Hispanic whites. In contrast, the National Epidemiologic Survey on Alcohol and Related Conditions (2001–2002) revealed African Americans had significantly higher PD rates than non-Hispanic whites, with 12-month prevalence rates of 16.6% for African Americans, 14.6% non-Hispanic whites, and 14.0% Hispanics [18].

The influence of race/ethnicity on perception and behavior is complex. First, there is substantial variability both within and between racial/ethnic groups. Second, environmental factors such as gender, socioeconomic status, level of discrimination/racism experienced, peer support, and acculturation also affect perception and behavior, and may be either confounded with or distinct from race/ethnicity [19,20,21,22]. For example, differences in self-reported psychological and physical health between African Americans and non-Hispanic whites are markedly reduced after accounting for income and, to a lesser extent, education [23]. Thus, race/ethnicity influences well-being via factors at both the individual (e.g., personal experiences of discrimination) and community (e.g., neighborhood resources) levels [24]. Racial/ethnic differences and the larger cultural context in which such differences are embedded influence not only the meaning that individuals ascribe to stressful experiences and how acceptable adaptive responses to stress are defined, but also how psychological symptoms of distress are expressed and how information about mental health disorders is understood [25,26]. However, investigating ethnic differences in personality pathology is in its infancy [6].

In one recent study, Ghafoori and Hierholzer [6] explored ethnic differences in personality pathology in a sample of 96 male combat veterans. In their review of the limited relevant literature, these authors note previous studies indicate higher rates of cluster A PD traits among African American veterans [26,27,28], with this difference potentially attributable to higher rates of ethnic discrimination [27]. However, in their sample, Hispanic male veterans had higher rates of cluster A PD traits (66.7%) than non-Hispanic white males (27.1%) and African American males (36.4%), and were more than four times as likely to have a cluster A PD, even after controlling for age, education, income, PTSD symptom severity, and level of combat exposure.

The higher prevalence of PTSD among women [29], the comorbidity of PTSD and PDs [4,5,6,7], and the increasing numbers of women in the military, particularly minority women [30], make it important to understand the relationships among these factors so that treatment needs can be identified and appropriate psychiatric services provided. To our knowledge, this is the first study examining PDs along ethnic/racial lines in a cohort of women veterans diagnosed with PTSD.

Our study expands on Ghafoori and Hierholzer’s [6] research by examining women veterans in particular, and also by reporting on the role of trauma-related covariates in PD cluster designation. Based on Ghafoori and Hierholzer’s [6] findings, we hypothesized Hispanic women in our sample would have higher rates of cluster A PDs after controlling for the covariates of age at treatment entry, marital status, combat exposure, childhood trauma, two or more traumas, sexual trauma, and current CAPS PTSD severity score. We also anticipated participants reporting childhood trauma would have an increased likelihood of cluster B PDs compared with participants reporting trauma experienced in adulthood only [4], as numerous studies report higher rates of borderline personality disorder (cluster B) among individuals reporting childhood abuse [4,31,32]. There is some evidence that both Hispanic and African American individuals may have higher rates of cluster B PD traits [26,33], and other evidence that childhood sexual abuse is associated with higher rates of cluster B PD traits in adulthood [34,35]. It is possible, then, that samples in which Hispanic and African American women have higher rates of cluster B PD traits are reflective of higher rates of childhood sexual abuse among those women. In a recent study of racial disparities in trauma exposure among women veterans, however, non-Hispanic white women were found to have experienced higher rates of childhood sexual abuse than African American women, although African American women were more likely to have experienced physical assault [36]. As such, we did not specifically hypothesize a link between ethnicity and cluster B PD traits, but again, did hypothesize that childhood trauma would be associated with higher rates of cluster B PD traits.

## 2. Method

### 2.1. Participants and Data Collection

Data were collected from 398 women veterans diagnosed with and receiving treatment for PTSD in the Women’s Stress Disorder Treatment Team (WSDTT) clinic at a VA facility located in the Southwest from 1995–2009. All WSDTT clinic patients are scheduled for the same psychological testing battery (including the Clinician-Administered PTSD Scale [CAPS] and the Millon Clinical Multiaxial Inventory-III [MCMI-III], evaluated in the present study) to confirm PTSD diagnoses and symptom severity and to assess for the presence of other psychopathology, such as personality disorder traits, that might impact treatment planning. However, for a variety of reasons, not all clinic patients complete this testing. Test completion is not a requirement for receiving treatment in the WSDTT clinic, and the clinic does not systematically assess differences between women who do and do not complete testing. The present sample represents the subset of women who both met the ethnicity inclusion criteria (described below) and completed testing. Out of the original 398 women in the clinic data set, 138 were excluded because they did not meet the ethnicity criteria (e.g., women identifying as biracial), or they had invalid, missing, or incomplete test data. The final subset consisted of 260 self-identified non-Hispanic white, Hispanic, and African American women veterans. Data for this study was collected through archival record review and approved by local VA and University of New Mexico Institutional Review Boards.

### 2.2. Instruments

*Clinician Administered PTSD Scale* (CAPS). The CAPS [37] is a 30-item structured clinical interview that corresponds to the DSM-IV criteria for PTSD [38,39] and is the gold standard in PTSD assessment. The CAPS was administered by staff psychologists or supervised psychology interns. The CAPS can be used to make a current (past month) or lifetime diagnosis of PTSD. The frequency and intensity of each symptom is rated on a 5-point ordinal scale. Inter-rater reliability is excellent, test-retest reliabilities are between 0.90 and 0.98 over one week, and there is a high degree of internal consistency with Cronbach’s alpha ranging from 0.73–0.85 [40]. For diagnostic purposes, a PTSD symptom was considered present if the frequency was rated as one (once a month) or higher and the intensity was rated two (moderate) or higher [41]. Internal consistency in the present sample of female veterans was computed using Cronbach’s alpha and found to be at acceptable levels (current CAPS = 0.89; lifetime CAPS = 0.86) and comparable to past research.

*Millon Clinical Multiaxial Inventory-III (MCMI-III)*. The MCMI-III [42] is a computerized psychological test, consisting of 175 true/false statements. The personality scales parallel the personality disorders of the DSM-III-R and DSM-IV, and items were chosen based on a theory of personality, with clinical norms. The normative sample was drawn from patients being treated or evaluated in mental health settings [43], and included some ethnic groups, 8.7% African Americans and 2.8% Hispanics [44]. Factor analysis supports the organization of the scales, and the internal consistency measures range from 0.66–0.90 with 20 of 26 scales exceeding 0.80 [45]. Raw scores are converted to base rate (BR) scores to allow comparison between the personality indices. Cutoff scores coordinated with disorder base rates provide more accurate diagnoses [46]. A BR greater than 84 indicates all the characteristics that define the disorder. BR scores between 75 and 84 indicate the presence of traits associated with the disorder, but below the diagnostic level. BR scores less than 75 are not considered diagnostically significant. Participants scoring 85 or above on the following scales were grouped as follows: paranoid, schizoid, and schizotypal PDs in cluster A; histrionic, narcissistic, antisocial, and borderline PDs, cluster B; and avoidant, dependent, and obsessive-compulsive PDs, cluster C.

### 2.3. Analytic Strategy

Non-Hispanic white, Hispanic, and African American were coded as dichotomous predictor variables in the regression models. The archival data review revealed five types of trauma which were categorized as follows: sexual trauma includes the sexual-trauma-only category and the combination category of sexual trauma plus other trauma; and non-sexual trauma includes the combat, physical/emotional, and other trauma categories. For age at trauma, the categories of childhood trauma and both childhood and adult trauma were combined as childhood trauma, such that age at trauma represents the earliest trauma experienced. This coding decision was made based on the fact that childhood trauma in particular has a cumulative effect [47]; for example, it is well-established that women who have experienced childhood sexual trauma are at increased risk for adult sexual trauma [48]. Demographic and clinical differences among ethnicities were assessed with Chi-square tests for categorical variables and analysis of variance (ANOVA) for continuous variables, with Fisher’s Least Significance Difference method of post hoc comparisons of means. We used binary logistic regression to predict positive PD cluster status in each of three clusters (A, B, C) for each of three ethnicities, with odds ratios and 95% confidence intervals (CI) reflecting the increased or decreased likelihood of positive cluster status. The first logistic regression analyses were univariate associations of positive PD clusters on ethnicity. Covariate variables were included in the second set of analyses to reduce potential confounding. We also conducted separate logistic regression analyses to determine whether positive PD clusters were associated with covariates (age at treatment entry, marital status, combat exposure, childhood trauma, two or more traumas, sexual trauma, and current CAPS PTSD severity score). If a positive PD cluster was significantly associated with an ethnicity, then each of the components of that PD cluster were examined post hoc to determine which components were involved in the finding. All tests were two-sided with significance set at 0.05. We concur with Osborne [49] who argues for not reporting negative (less than 1.0) odds ratios, so we do not discuss negative odds ratios.

## 3. Results

### 3.1. Descriptive Statistics

Of 260 women veterans, 64.2% self-identified as non-Hispanic white, 27.3% Hispanic, and 8.5% African American. The mean age of participants was 43 (range 19–70); and 28% reported being married. These women served largely in the Army (42%), Air Force (30%), and Navy (20%), and 16% reported being deployed to a combat zone (combat exposure). Multiple traumas were experienced by most (88%), and, 93% reported sexual trauma only or sexual trauma plus other trauma during the CAPS assessment. In terms of PDs, 44.6% (*n* = 116) met criteria for a cluster A PD, 22.7% (*n* = 59) for cluster B, and 54.2% (*n* = 141) for cluster C. Some participants had more than one PD.

Table 1 below presents the percentage by ethnicity of veterans meeting diagnostic criteria for a PD. Although the antisocial PD scale trended toward significance (*p* = 0.054) this result was likely due to small numbers (non-Hispanic white, *n* = 11, Hispanic, *n* = 1, African American, *n* = 3) meeting the antisocial PD criteria. There were no significant differences when we compared those meeting diagnostic criteria for a PD to those who did not meet criteria.

**Table 1:** Percentage meeting Millon Clinical Multiaxial Inventory-III (MCMI-III) diagnostic criteria for a personality disorder by ethnicity and cluster.

	Non-Hispanic White		Hispanic		African American		
	(*n* = 167)		(*n* = 71)		(*n* = 22)		
	*n*	%	*n*	%	*n*	%	*p*
**Cluster A**							
Paranoid PD	10	6.0	7	9.9	4	18.2	0.09
Schizoid PD	62	37.1	21	29.6	9	40.9	0.48
Schizotypal PD	20	12.0	9	12.7	5	22.7	0.36
**Cluster B**							
Borderline PD	28	16.8	9	12.7	5	22.7	0.48
Antisocial PD	11	6.6	1	1.4	3	13.6	0.05
Narcissistic PD	8	4.8	4	5.6	3	13.6	0.21
Histrionic PD	4	2.4	4	5.6	2	9.1	0.13
**Cluster C**							
Obsessive-Compulsive PD	19	11.4	6	8.4	6	27.3	0.07
Avoidant PD	66	39.5	22	31.0	8	36.4	0.47
Dependent PD	45	26.9	11	15.5	4	18.2	0.14

Note: Diagnostic criteria = base rate score on MCMI-III greater than 84; PD = Personality Disorder.

### 3.2. Relationships Among Demographic and Covariate Characteristics by Ethnicity (Table 2)

There were no significant differences among ethnic groups on marital status, Armed Forces branch, age at trauma, number of traumas, or trauma type. However, Hispanics were significantly younger (*M* = 40.9, *SD* = 10.9) than non-Hispanic whites (*M* = 46.4, *SD* = 10.5, *p* < 0.001) at entry into treatment for PTSD; and were significantly more likely to have experienced combat exposure than non-Hispanic whites (28.2% and 12.6%, respectively, *p* = 0.05). Current CAPS PTSD severity scores revealed no significant differences among ethnic groups. Likewise, there were no significant differences in PD clusters among ethnic groups.

### 3.3. Associations among PD Clusters and Ethnicities (Table 3)

There were no significant differences among ethnic groups in the non-adjusted or “crude” odds ratio rates, although African Americans were trending toward significance for more frequent cluster A PDs (*p* = 0.07). After adjusting for age at treatment entry, marital status, combat exposure, childhood trauma, two or more traumas, sexual trauma, and current (past month) CAPS PTSD severity score, the odds of having a cluster A PD were almost three times greater (*p* = 0.046) for African American women veterans. Ethnicity was not associated with PD clusters B or C.

**Table 2:** Demographic and covariate characteristics by ethnicity in a group of women veterans diagnosed with Post Traumatic Stress Disorder (PTSD).

	Non-Hispanic White (*n* = 167)		Hispanic (*n* = 71)		African American (*n* = 22)	
	Mean	SD	Mean	SD	Mean	SD
Age at treatment entry †	46.4^A^	10.5	40.9^B^	10.9	42.6^AB^	7.9
Current CAPS PTSD severity score	74.2	23.9	73.1	25.5	73.4	28.1
	*n*	%	*n*	%	*n*	%
Marital status						
Married/cohabitating	47	28.1	19	26.8	8	36.4
Single/divorced/separated/widowed	120	71.9	52	73.2	14	63.6
Armed forces branch						
Army	70	41.9	34	47.9	5	22.7
Navy	32	19.2	15	21.1	6	27.3
Air Force	52	31.1	15	21.1	11	50.0
Marine Corps/Coast Guard/Reserves	13	7.8	7	9.9	0	0.0
Combat exposure **‡**						
Yes	21	12.6^B^	20	28.2^A^	2	9.1^AB^
No	146	87.4	51	71.8	20	90.9
Age at trauma						
Childhood (<age 18)	14	8.4	6	8.4	1	4.5
Adult	59	35.3	30	42.2	6	27.3
Both childhood and adult	94	56.3	35	49.3	15	68.2
Number of traumas						
One	22	13.2	7	9.9	3	13.6
Two or more	145	86.8	64	90.1	19	86.4
Trauma type						
Sexual-trauma-only	78	46.7	24	33.8	13	59.1
Sexual trauma + other trauma	77	46.1	41	57.7	9	40.9
Non-sexual trauma *	12	7.2	6	8.4	0	0.0
Personality Disorder Clusters **						
Cluster A	73	43.7	29	40.8	14	63.6
Cluster B	38	22.7	14	19.7	7	31.8
Cluster C	96	57.5	33	46.5	12	54.5

Note, CAPS = Clinician Administered PTSD Scale. PTSD = posttraumatic stress disorder. Current CAPS = past month; † ANOVA, *p*<0.001; ‡ Chi square, *p*<0.001; ^A,B^ Designations: different letters indicate significant differences among groups in post-hoc testing (*p*<0.05). * Non-sexual trauma includes combat, physical/emotional, motor vehicle crash, natural disaster. ** Percentages do not equal 100 as some participants had more than one personality disorder.

**Table 3:** Associations among personality disorder clusters and ethnicities in a group of women veterans diagnosed with PTSD.

	Personality Disorder Clusters					
Cluster A			Cluster B		Cluster C	
Ethnicity	Crude OR	Adjusted ^a^ OR	Crude OR	Adjusted ^a^ OR	Crude OR	Adjusted ^a^ OR
	(95% CI)	(95% CI)	(95% CI)	(95% CI)	(95% CI)	(95% CI)
Non-Hispanic Whites	0.90(0.54,1.50)	0.77(0.44,1.36)	1.01(0.55,1.85)	0.97(0.51,1.84)	1.44(0.87,2.40)	1.23(0.71,2.12)
	*p* = 0.69	*p* = 0.37	*p* = 0.97	*p* = 0.92	*p* = 0.16	*p* = 0.46
Hispanics	0.81(0.47,1.41)	0. 90(0.49,1.66)	0.79(0.40,1.54)	0.88(0.43,1.79)	0.65(0.38,1.13)	0.83(0.46,1.50)
	*p* = 0.45	*p* = 0.74	*p* = 0.48	*p* = 0.72	*p* = 0.12	*p* = 0.53
African Americans	2.33(0.94,5.77)	2.77(1.02,7.52)	1.67(0.65,4.31)	1.43(0.52,3.91)	1.01(0.42,2.44)	0.88(0.35,2.21)
	*p* = 0.07	*p* = 0.046	*p* = 0.29	*p* = 0.48	*p* = 0.97	*p* = 0.79

Note, Cluster A = Paranoid, Schizoid, and Schizotypal PDs. Cluster B = Antisocial, Borderline, Histrionic, and Narcissistic PDs. Cluster C = Avoidant, Dependent, and Obsessive-Compulsive PDs. Crude = unadjusted. OR = odds ratio. CI = confidence intervals. ^a^ Adjusted for age at treatment entry, marital status, combat exposure, childhood trauma, two or more traumas, sexual trauma, and current CAPS PTSD score. Childhood and both childhood and adult trauma were combined as childhood trauma. Sexual-trauma-only and sexual trauma plus other trauma were combined as sexual trauma.

### 3.4. Additional Analysis

Paranoid, schizoid, and schizotypal PDs comprise cluster A, and we assessed whether African American ethnicity contributed to any one of these three PDs in the cluster before and after adjusting for covariates. Neither the non-adjusted or adjusted analyses revealed a significant association; however, the Paranoid PD scale trended toward significance (*p* = 0.08). These marginal findings are probably due to small numbers (paranoid, *n* = 4; schizoid, *n* = 9; schizotypal, *n* = 5).

**Table 4:** Associations among personality disorder clusters and covariates and in a group of women veterans diagnosed with PTSD.

	Personality Disorder Clusters		
	Cluster A	Cluster B	Cluster C
	Odds Ratio (95% CI)	Odds Ratio (95% CI)	Odds Ratio (95% CI)
Age at treatment entry	1.01(0.98,1.03)	0.98(0.96,1.01)	1.01(0.99,1.04)
	*p* = 0.55	*p* = 0.29	*p* = 0.22
Marital status	1.46(0.85,2.51)	1.54(0.83,2.86)	1.57(0.91,2.73)
	*p* = 0.17	*p* = 0.17	*p* = 0.11
Combat exposure	0.61(0.31,1.22)	0.62(0.26,1.47)	0.39(0.20,0.77)
	*p* = 0.16	*p* = 0.27	*p* = 0.006
Childhood trauma	0.64(0.39,1.07)	1.94(1.01,3.72)	0.97(0.58,1.61)
	*p* = 0.09	*p* = 0.046	*p* = 0.90
Two or more traumas	0.44(0.20,0.93)	1.05(0.43,2.58)	0.68(0.32,1.45)
	*p* = 0.03	*p* = 0.91	*p* = 0.32
Sexual trauma	1.11(0.68,1.82)	1.41(0.79,2.53)	2.10(1.27,3.47)
	*p* = 0.67	*p* = 0.25	*p* = 0.004
Current CAPS PTSD score	1.51(1.25,1.81)	1.30(1.05,1.60)	1.20(1.01,1.42)
	*p* = 0.0001	*p* = 0.02	*p* = 0.040

Note, CI = confidence intervals. Childhood and both childhood and adult trauma were combined as childhood trauma. Sexual-trauma-only and sexual trauma plus other trauma were combined as sexual trauma.

### 3.5. Associations among PD Clusters and Covariates (Table 4)

The odds of having a cluster B PD were almost two times greater in women with childhood trauma than in women reporting adult trauma only (*p* = 0.046). The odds of having a cluster C PD were two times greater in women reporting sexual trauma than in women reporting non-sexual trauma (*p* = 0.004). The higher the current CAPS PTSD severity score, the greater the odds of having a cluster A (*p* = 0.0001), cluster B (*p* = 0.02), or cluster C (*p* = 0.04) PD. Two covariates were significantly associated with reduced risk of PD clusters. First, the odds of having a cluster A PD were less than half as high in women reporting two or more traumas than in women reporting only one trauma (*p* = 0.03). Second, the odds of having a cluster C PD were also less than half as high in women reporting combat exposure (*p* = 0.006).

## 4. Discussion

The intention of this study was to expand on Ghafoori and Hierholzer’s [6] study by investigating ethnic differences in PDs among women veterans with PTSD. The only significant ethnic difference in our findings was that African American women veterans had a higher odds of having cluster A PD, whereas Ghafoori and Hierholzer reported that Hispanics were more likely to have cluster A PDs then non-Hispanic whites. A closer look at the data to investigate whether ethnicity contributed to one of the specific PDs in cluster A revealed no significant contribution, with the Paranoid PD scale only trending toward significance. The majority of studies of PDs do not include ethnicity, and studies reporting PDs among African Americans have been inconsistent. This is an important area of discussion and there is no definitive support in the literature for African Americans having higher rates of cluster A PDs. However, we offer some possible explanations for our findings.

First, the MCMI-III may not be sensitive to variations in cultural thought and behavior patterns [6]. Though the MCMI-III is widely used, there are concerns with regard to its continued use with minority populations [50], primarily because of the underrepresentation of minorities in the standardization samples [51]. This is not a small matter, considering the growing minority population in the U.S. [52]. On the other hand, the normative sample for the MCMI-III included almost 9% African Americans [44]. Importantly, studies have revealed African Americans tend to score higher than non-Hispanic whites on MCMI scales [53], and particularly the Paranoid PD scale [28].

Second, culture influences the expression of problems [26], and, by extension, the diagnosis of psychopathology. Paranoid PD is characterized by a generalized suspicion and mistrust of others. African American women scoring higher on measures of paranoia and hence cluster A PDs may reflect an adaptive response to a history of racial discrimination and oppression [54,55], and may not necessarily represent pathological responding in the face of being in the minority culture. For example, the paranoid scale may capture a realistic preoccupation with personal rights and a heightened sense of these being violated.

Third, although not specific to ethnicity but clouding the diagnostic picture, is the overlap between cluster A schizoid and schizotypal traits and PTSD symptoms [56]. For example, dissociative episodes such as flashbacks (a PTSD symptom) could lead to endorsing items that are schizotypal traits such as questions about unusual perceptual experiences. Additionally, “markedly diminished interest or participation in significant activities” is a PTSD symptom that might lead individuals to endorse “taking pleasure in few if any activities” which is a schizoid trait. Furthermore, for those with PTSD related to sexual trauma, the schizoid trait of “has little, if any, interest in having sexual experiences with another person” may have an increased probability of being endorsed [57].

We also evaluated relationships among PDs and covariates, and found women with sexual trauma were twice as likely to have a cluster C PD, characterized by anxious or fearful behavior, than women with non-sexual trauma. These results differ from others who have reported increased likelihood of borderline personality disorder (cluster B) with sexual trauma [32]. Our findings may be a consequence of the population under study and the maladaptive fear structures of those with PTSD (see Foa and Kozak’s Emotional Processing Theory [58]).

We did confirm our hypothesis that those with childhood trauma would have an increased likelihood of cluster B PDs compared to women reporting trauma only in adulthood. Cluster B PDs include borderline personality disorder (BPD), and our finding is consistent with other research findings of childhood trauma and BPD [34,35]. This is important information for clinicians treating women veterans as there is evidence of greater pathology and poorer outcomes when PTSD is comorbid with BPD [5,59,60].

While this study expands our understanding of ethnic differences in PDs among women veterans with PTSD, several limitations should be considered, including the cross-sectional design of the study, the reliance on self-report measures, and the instrument used to measure personality disorders, MCMI-III, not being validated separately on African American and Hispanic populations, raising overpathologizing as a concern until diagnostic validity is established. Notably, we did not have information on comorbid disorders that may influence assessment responses [3], and we did not have any information on exposure to racism (e.g., severity and duration [61]) that might have shaped assessment responses. The possibility that racism and oppression may play a specific role in the relationship between African American ethnicity and cluster A PDs should be explored, given African American women veterans in our sample had a nearly three times greater likelihood of having a Cluster A PD in an adjusted odds ratio. Also, generalizability of the results is limited as the study population is women veterans seeking treatment for PTSD at one VA facility. However, this real-world clinical sample did show differences among ethnic groups.

## 5. Conclusions

Trauma is defined as experiencing, witnessing, or being confronted with an event or events that involved actual or threatened death or serious injury, or a threat to the physical integrity of self or others, and trauma can and does lead to PTSD [62] and PDs [4,6]. We also know that ethnicity, and correlates of ethnic differences such as cultural norms and socioeconomic circumstances, both impact behaviors assessed when diagnoses of psychopathology are made, including diagnoses of both PTSD [61] and personality disorders [17,18,25].While there are studies that hint at differences among ethnicities with regard to PDs [6,26,28], there have been no definitive studies elucidating the role of PTSD and ethnicity in understanding PDs. The extant literature and our findings establish the need for further investigation to clarify relationships among cultural influences (e.g., ethnicity), PTSD, and PDs, using instruments with diagnostic validity for minority populations to reduce or eliminate any diagnostic false positives, documenting comorbid disorders, and assessing for exposure to racism. The influence of diagnoses on treatment planning and, therefore, treatment outcomes, cannot be overemphasized.
